# Theoretical studies on the intramolecular cyclization of 2,4,6-*t*-Bu_3_C_6_H_2_P=C: and effects of conjugation between the P=C and aromatic moieties

**DOI:** 10.3762/bjoc.10.103

**Published:** 2014-05-07

**Authors:** Masaaki Yoshifuji, Shigekazu Ito

**Affiliations:** 1 Department of Chemistry, Graduate School of Science, Tohoku University, Aoba, Sendai 980-8578, Japan; 2 Department of Applied Chemistry, Graduate School of Science and Engineering, Tokyo Institute of Technology, Meguro, Tokyo 152-8552, Japan

**Keywords:** 3,4-dihydro-1-phosphanaphthalene, intramolecular cyclization, organophosphorus, phosphanylidenecarbene, steric protection, theoretical calculation

## Abstract

The intramolecular C–H insertion of the Mes*-substituted phosphanylidenecarbene [Mes*P=C:] (Mes* = 2,4,6-*t*-Bu_3_C_6_H_2_) and physicochemical properties of the cyclized product, 6,8-di-*tert-*butyl-3,4-dihydro-4,4-dimethyl-1-phosphanaphthalene were studied based on ab initio calculations. Whereas the alternative Fritsch–Buttenberg–Wiechell-type rearrangement requires almost no activation energy, the intramolecular cyclization needs an activation energy of 12.3 kcal/mol at the MP2(full)/6-31G(d) condition. DFT calculations supported that the optimized structure of the cyclization product of Mes*P=C: suggests remarkable conjugation effects between the nearly coplanar P=C skeleton and the aryl moiety.

## Introduction

**Scheme 1:**
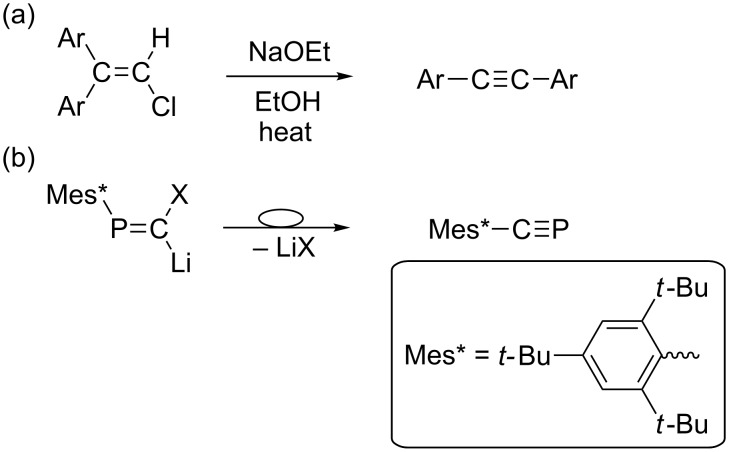
(a) The original FBW rearrangement reaction and (b) the phosphorus version of FBW rearrangement.

Sterically demanding groups on the phosphorus atom play an important role in the chemistry of low-coordinate phosphorus compounds and the supermesityl (Mes* = 2,4,6-tri-*tert*-butylphenyl) group was successfully applied to stabilize and characterize a diphosphene (Mes*P=PMes*) for the first time [1]. The effect of the Mes* group on the stabilization of various kinds of unusual phosphorus compounds has been clarified so far [2–4]. Phosphanylidenecarbene [RP=C:], a heavier congener of alkylidenecarbene (phosphaisonitrile) has been an intriguing reaction intermediate containing a low-coordinated phosphorus atom, and afforded a number of unique organophosphorus compounds [5–6]. The phosphorus version of Fritsch–Buttenberg–Wiechell (FBW) reaction [7–10] of Mes*P=C(X)Li (X = halogen, Mes* = 2,4,6-*t*-Bu_3_C_6_H_2_) affording an air-stable phosphaalkyne Mes*C≡P is a typical example for understanding the chemistry of a phosphanylidenecarbenoid (Scheme 1) [11–13]. The phosphorus version of FBW rearrangement showed considerable stereospecificity in affording phosphaalkyne, which could be explicable by plausible reaction mechanisms including formation of the phosphavinyl anion intermediate without generation of phosphanylidenecarbene [10,14].

**Scheme 2:**
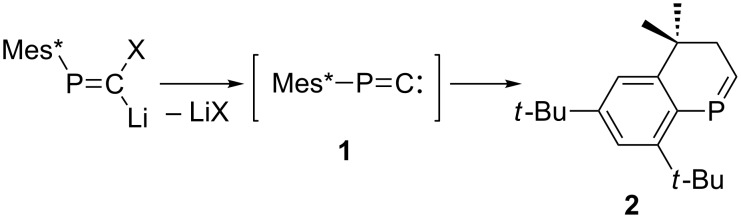
Intramolecular C–H insertion of phosphanylidenecarbene.

As an alternative reaction of phosphanylidenecarbenoid, we have previously found the intramolecular cyclization reaction affording 6,8-di-*tert-*butyl-3,4-dihydro-4,4-dimethyl-1-phosphanaphthalene (**2**) putatively through formation of phosphanylidenecarbene **1** generated from Mes*P=C(Br)Li (Scheme 2) [15]. In contrast to the selective formation of Mes*C≡P from (*E*)-Mes*P=C(Cl)Li [11–12], facile removal of the bromide ion in Mes*P=C(Br)Li might be critical for the C–H insertion. The C–H insertion of carbene has been studied well [16], and thus intensive studies on the intramolecular cyclization of **1** would be necessary to develop the chemistry of reactive intermediates containing low-coordinated heavier main group elements [17]. Additionally, the structure of **2** is expected to be quite unique as the P=C π-system is nearly coplanar with the aromatic ring. In our previous paper, unique photo-absorption properties of **2** were discussed in comparison with the Mes*-substituted phosphaalkenes where the P=C and the Mes* aryl moieties are almost perpendicular [14].

## Results and Discussion

In this paper we discuss the intramolecular cyclization of **1** and the structural aspects of **2** based on theoretical calculation data. Ab initio and DFT calculations were carried out with the Gaussian 09 program package [18].

**Figure 1:**
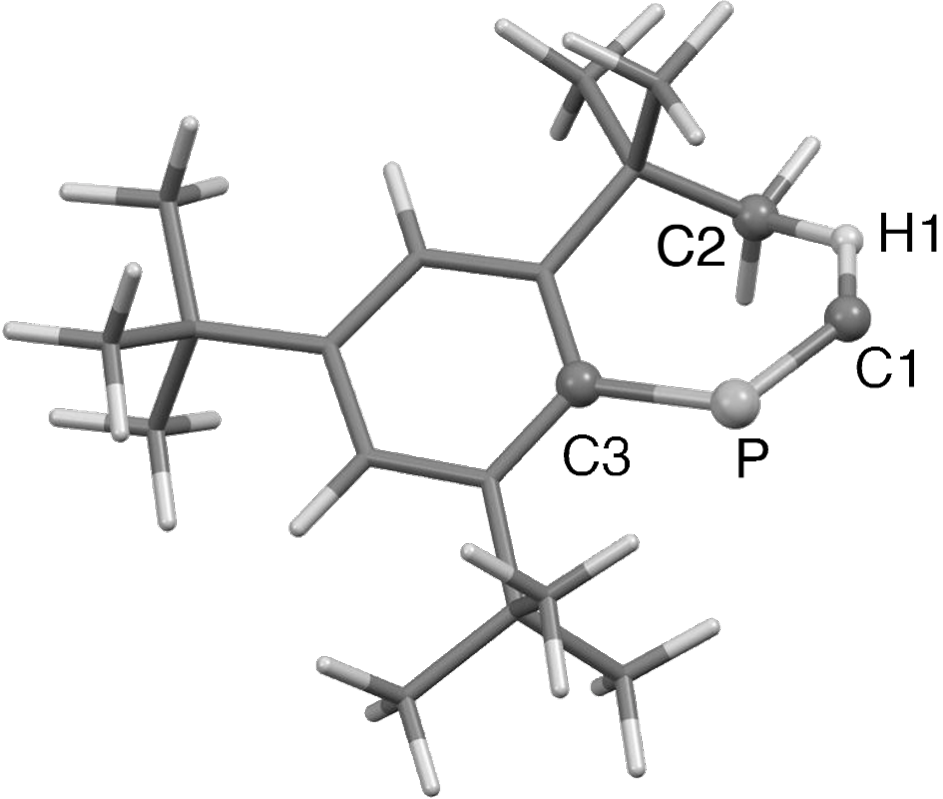
Optimized structure of the transition state (TS) for the intramolecular C–H insertion of **1** [MP2(Full)/6-31G(d)]. Bond lengths (Å): P–C1 1.660, C1–H1 1.228, C2–H1 1.281, P–C3 1.865.

**Figure 2:**
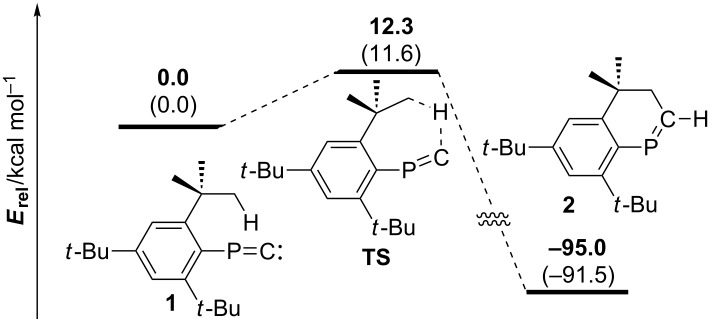
Computationally characterized cyclization procedures of **1** affording **2** [MP2(full)/6-31G(d)]. Values in boldface correspond to relative energies (kcal/mol). Values in parentheses display Gibbs free energies (*G*, kcal/mol at 298.15 K).

**Figure 3:**
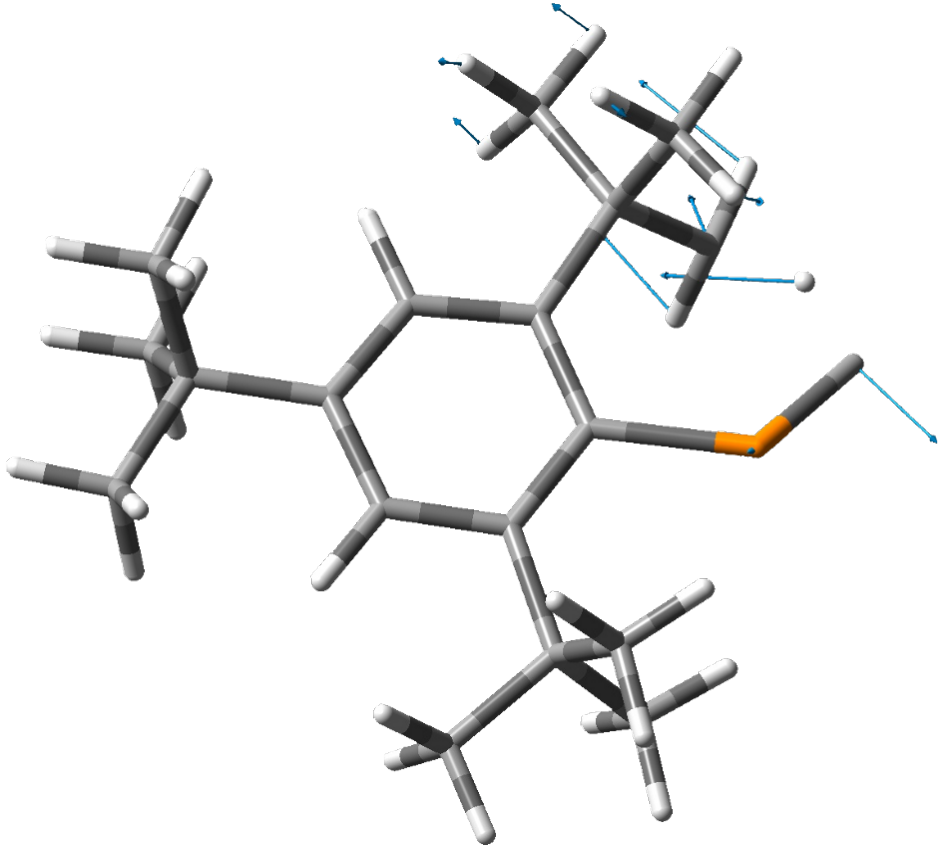
Displacement vectors of the transition state (ν = 216.93 *i* cm^–1^).

Structures of **1** in the singlet state and **2** were optimized at the MP2(Full)/6-31G(d) level, and subsequently employed for calculation of the transition state. DFT methods were avoided in this calculation, as the Mes*–P–C angle was considerably bent at the level, such as B3LYP/6-31G(d) [14]. The bent structure optimized by the DFT method might reflect overestimation of the sp^2^-type hybridization of the phosphorus atom because of the sterically encumbered Mes* group [19]. Figure 1 displays the DFT-optimized structure of the transition state (TS), and Figure 2 shows the energy profile of the cyclization process. Considerable elongation of the C–H bond of the corresponding methyl group has been characterized, whereas the P=C length was found comparable to that in **1** (vide infra). The optimized activation energy (Δ*E*_a_) was 12.3 kcal/mol, and the Gibbs free energy Δ*G* was estimated as 11.6 kcal/mol. Such energy profile indicates a sharp contrast to the modeled FBW rearrangement of phosphanylidenecarbene requiring no activation energy [20], and would be partially explicable for the experimental result that the phosphanylidenecarbenoid [Mes*P=C(Br)Li] afforded both phosphaalkyne [Mes*C≡P] and **2** [15]. The single imaginary frequency was optimized for the transition state (Figure 3).

**Figure 4:**
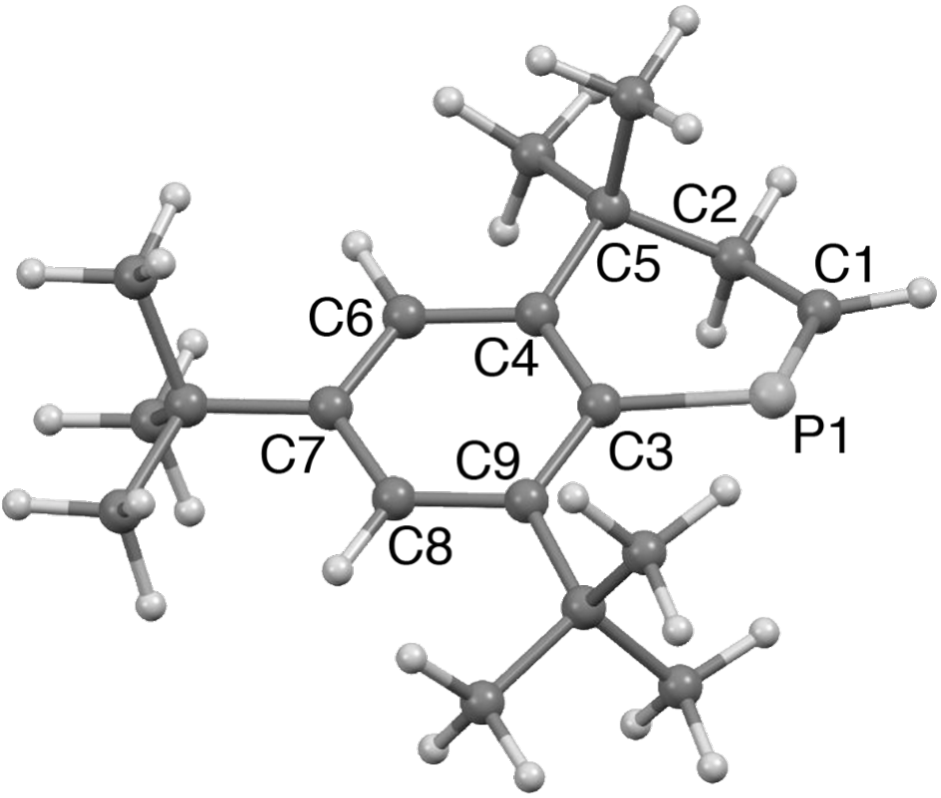
Optimized structure of **2** [MP2(full)/6-31G(d)]. Bond distances (Å): P1–C1 1.678, C1–C2 1.491, P1–C3 1.840, C2–C5 1.534, C3–C4 1.424, C4–C5 1.530, C4–C6 1.396, C6–C7 1.394 C7–C8 1.393, C8–C9 1.403, C3–C9 1.427. Bond angle and dihedral angles (°): C3–P1–C1 101.7, C1–P1–C3–C4 22.9, C1–P1–C3–C9 160.1.

Figure 4 displays the optimized structure of **2** [MP2(full)/6-31G(d)]. Relative energy (*E*_rel_) and Gibbs free energy (*G*) of **2** to **1** were determined as 95.0 kcal/mol and 91.5 kcal/mol, respectively. Whereas the P1–C1 distance is typical for phosphaalkenes [21], dihedral angle of the P=C and almost planar benzene ring is close to co-planar due to the fused 6-membered ring [*τ*(C1–P1–C3–C4] = 22.9°, *τ*(C1–P1–C3–C9) = 160.1°]. Steric encumbrance causes elongation of the C–C bonds of C3–C4 and C8–C9.

**Figure 5:**
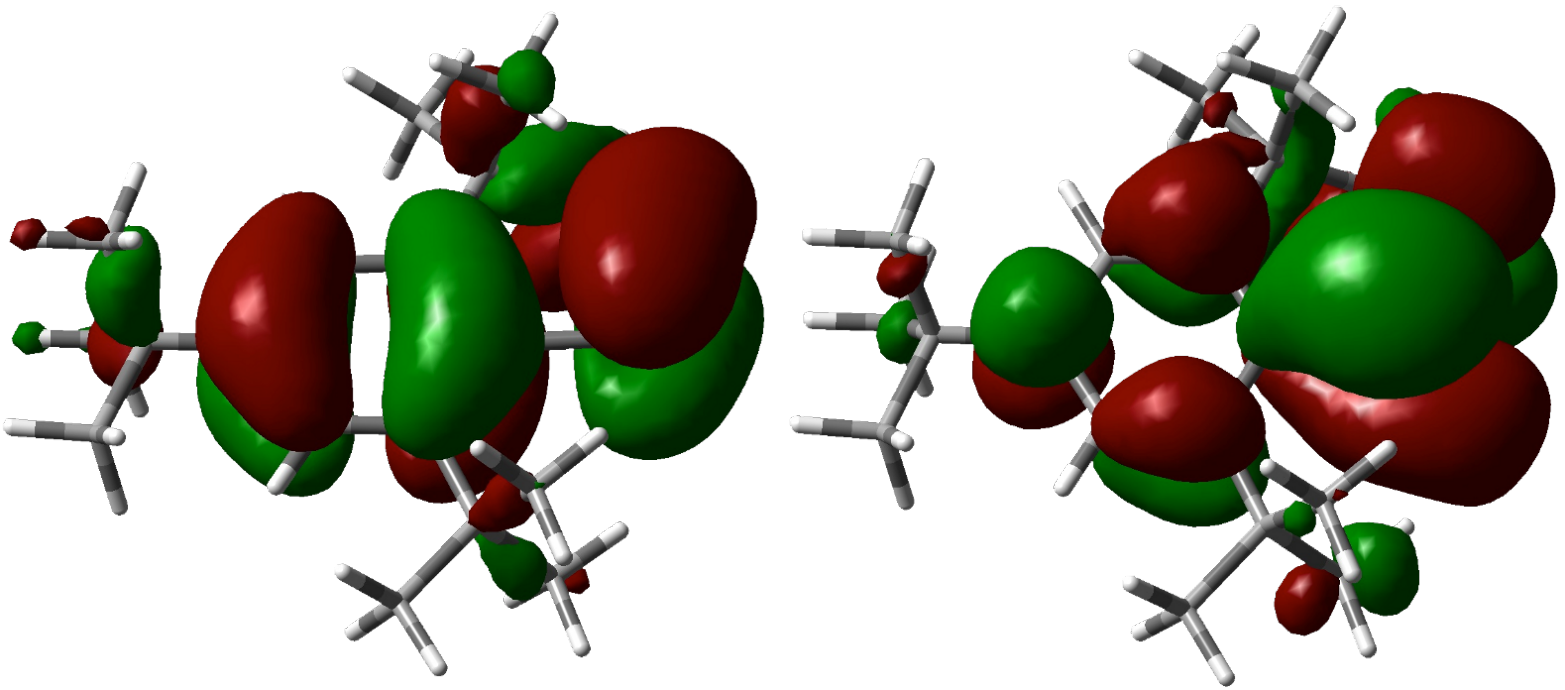
HOMO (left) and LUMO (right) of **2**.

Except for such as phosphinines (or phosphabenzenes), **2** would be one of key compounds that are available for understanding the conjugation effect between the heavier π-system and the aromatic moiety. The P=C skeleton of **2** would interact with the nearly coplanar benzene ring, and indeed, the UV absorption spectra exhibited a large absorption coefficient in comparison with the Mes*-substituted phosphaalkene. The HOMO and LUMO orbitals of **2** indicate remarkable contribution of the benzene ring for conjugation with the P=C π-system (Figure 5). The TD-SCF calculation of **2** using CAM-B3LYP/DGDZVP conditions characterized the HOMO–LUMO transition at 289 nm with a relatively large absorption coefficient (*f* = 0.162). On the other hand, the absorption maximum of **2** was slightly blue-shifted in comparison with that of the Mes*-substituted phosphaalkenes, which corresponded the TD-SCF calculation of Mes*P=CH_2_ determining absorption at 292 nm. In the case of Mes*P=CH_2_, the HOMO orbital is composed of the lone pair of the phosphorus, which corresponds to the weak absorption (*f* = 0.0139) [15] (see also Supporting Information File 1).

## Conclusion

In conclusion, the chemistry of the intramolecular C–H insertion of phosphanylidenecarbene **1** affording **2** was studied by ab initio and DFT calculations. The intramolecular cyclization requires an activation energy, whereas the phosphorus version of the FBW rearrangement proceeded without an energetic barrier. The optimized structure of **2** indicates the possible conjugation between the P=C π-system and aromatic substituent, which induces remarkably different physicochemical properties for the Mes*-substituted phosphaalkenes, where the P=C moiety is almost perpendicular to the aromatic plane.

## Supporting Information

File 1 UV Spectra for **2** and Mes*P=C(H)Me and MO for **2** and Mes*P=CH_2_.

File 2 Calculation data for **1**, **TS**, **2**, Mes*P=CH_2_ and [MeP=C:].
