# Supplementary material for: Theoretical studies on the intramolecular cyclization of 2,4,6-t-Bu3C6H2P=C: and effects of conjugation between the P=C and aromatic moieties
Source: Beilstein J Org Chem. 2014 May 7;10:1032–6. doi: 10.3762/bjoc.10.103 (PMC4077427; doi:10.3762/bjoc.10.103)
Supplement: File 1 — UV Spectra for 2 and Mes*P=C(H)Me and MO for 2 and Mes*P=CH2. [file Beilstein_J_Org_Chem-10-1032-s001.pdf]

Supporting Information

for

Theoretical studies on the intramolecular cyclization of 2,4,6-*t*-$Bu_3C_6H_2P$=C: and effects of conjugation between the P=C and aromatic moieties

Masaaki Yoshifuji[1]* and Shigekazu Ito[1,2]

Address: [1]Department of Chemistry, Graduate School of Science, Tohoku University, Aoba, Sendai 980-8578, Japan and [2]Present address: Department of Applied Chemistry, Graduate School of Science and Engineering, Tokyo Institute of Technology, Meguro, Tokyo 152-8552, Japan

Email: Masaaki Yoshifuji - yoshifj@m.tohoku.ac.jp

* Corresponding author

UV Spectra for 2 and Mes*P=C(H)Me and MO for 2 and Mes*P=CH_2

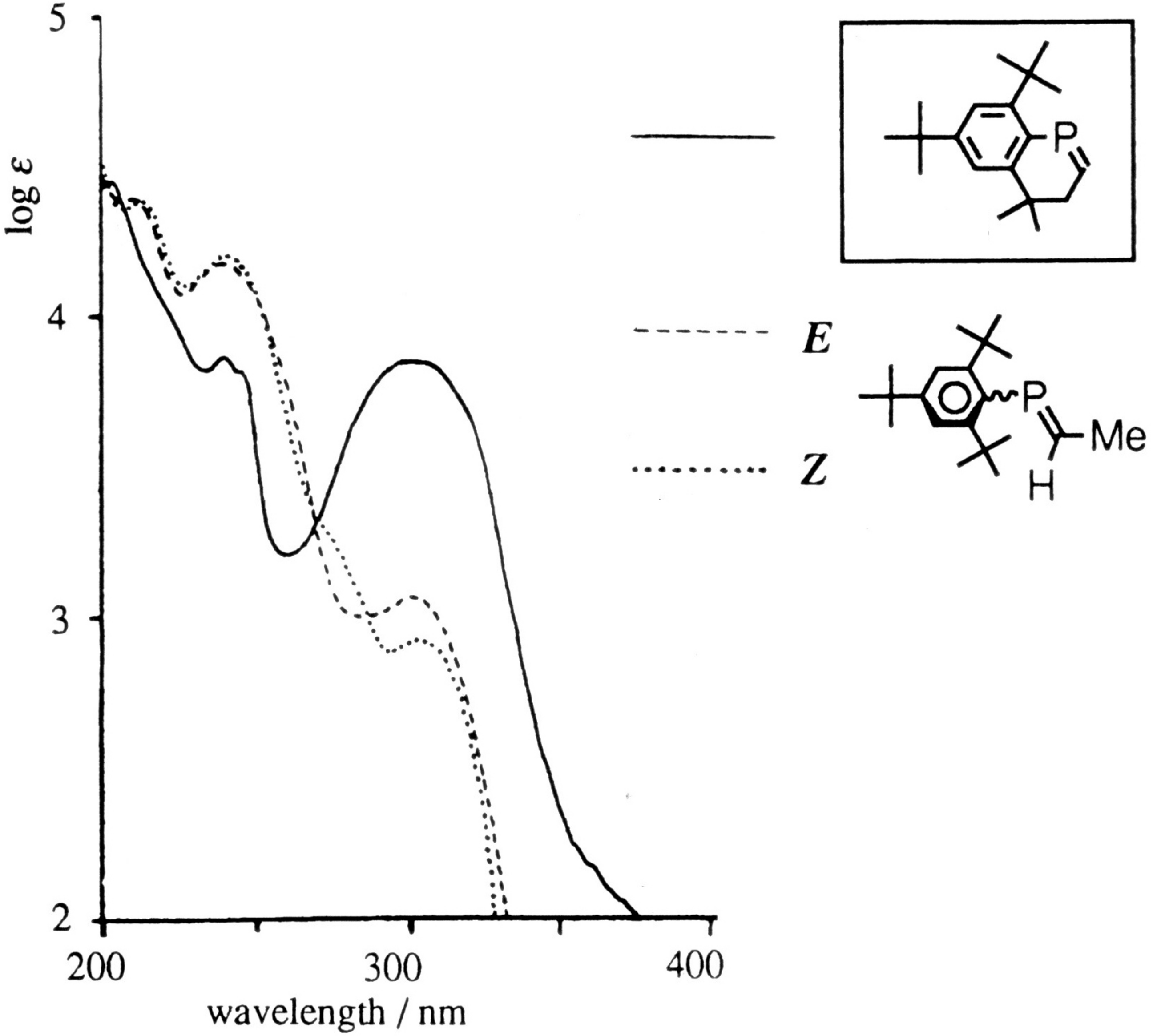

5
4
3
2
log ε
200
300
400
wavelength / nm
P
E
Z
P
Me
H

Molecular Orbital: Phosphaalkene

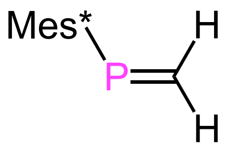

LUMO
–0.180 eV

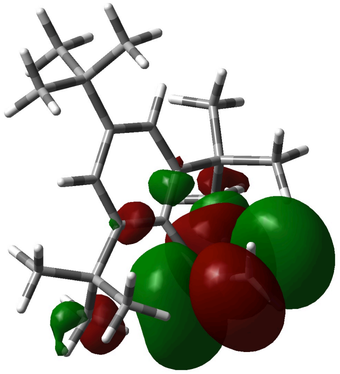

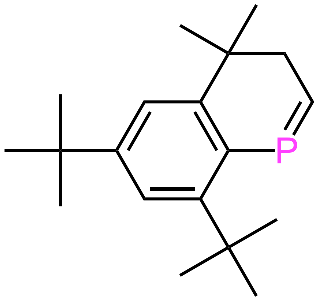

LUMO
–0.422 eV

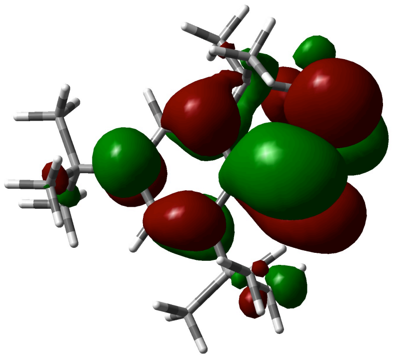

HOMO
–7.64 eV

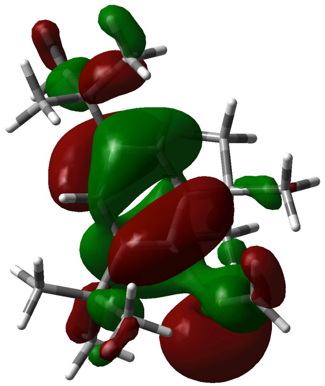

HOMO
–7.34 eV

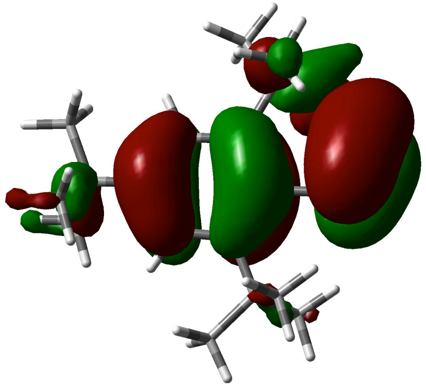

HOMO–1
–7.95 eV

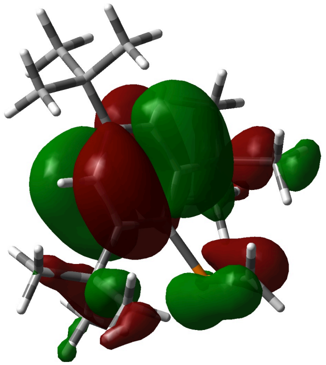

HOMO–1
–8.01 eV

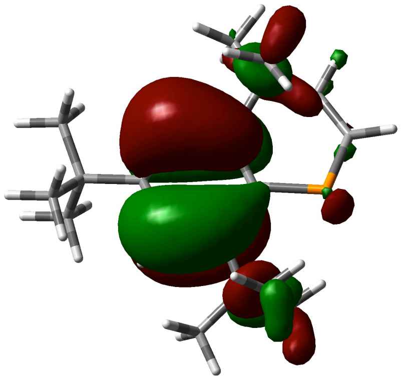

CAM-B3LYP/DGDZVP
