# Supplementary material for: Theoretical studies on the intramolecular cyclization of 2,4,6-t-Bu3C6H2P=C: and effects of conjugation between the P=C and aromatic moieties
Source: Beilstein J Org Chem. 2014 May 7;10:1032–6. doi: 10.3762/bjoc.10.103 (PMC4077427; doi:10.3762/bjoc.10.103)
Supplement: File 2 — Calculation data for 1, TS, 2, Mes*P=CH2 and [MeP=C:]. [file Beilstein_J_Org_Chem-10-1032-s002.pdf]

Supporting Information

for

Theoretical studies on the intramolecular cyclization of 2,4,6-*t*-$Bu_3C_6H_2P{=}C$: and effects of conjugation between the P=C and aromatic moieties

Masaaki Yoshifuji[1]* and Shigekazu Ito[1,2]

Address: [1]Department of Chemistry, Graduate School of Science, Tohoku University, Aoba, Sendai 980-8578, Japan and [2]Present address: Department of Applied Chemistry, Graduate School of Science and Engineering, Tokyo Institute of Technology, Meguro, Tokyo 152-8552, Japan

Email: Masaaki Yoshifuji - yoshifj@m.tohoku.ac.jp

* Corresponding author

Calculation data for 1, TS, 2, Mes*P=CH_2 and [MeP=C:]

Mes*P=C: (**1**)

mp2=full/6-31g(d)

EUMP2 = -1079.6426478013 A.U.

Thermal correction to Gibbs Free Energy = 0.391280

Center Number	Atomic Number	Atomic Type	Coordinates (Angstroms) X	Y	Z
1	15	0	2.791074	0.093184	-0.000484
2	6	0	4.431194	0.159246	-0.001088
3	6	0	0.967492	0.021430	-0.000037
4	6	0	0.329487	-1.250376	-0.000010
5	6	0	0.231501	1.242436	0.000000
6	6	0	-1.072350	-1.252041	-0.000097
7	6	0	-1.161756	1.130053	-0.000081
8	6	0	-1.838623	-0.090077	-0.000187
9	1	0	-1.584735	-2.201454	-0.000136
10	1	0	-1.754014	2.034586	-0.000112
11	6	0	1.066305	-2.603327	0.000240
12	6	0	0.860477	2.648307	0.000235
13	6	0	1.698843	2.871963	1.268842
14	1	0	2.568359	2.216859	1.330435
15	1	0	2.068340	3.903127	1.285017
16	1	0	1.083289	2.714690	2.160386
17	6	0	1.698391	2.872699	-1.268538
18	1	0	2.067909	3.903864	-1.284222
19	1	0	2.567854	2.217599	-1.330843
20	1	0	1.082503	2.715986	-2.159951
21	6	0	-0.208755	3.749845	0.000748
22	1	0	-0.843039	3.710810	-0.889967
23	1	0	-0.843031	3.709960	0.891430
24	1	0	0.296272	4.720238	0.001189
25	6	0	1.919820	-2.761069	-1.268254
26	1	0	2.734982	-2.039482	-1.330407
27	1	0	2.369436	-3.759917	-1.283856
28	1	0	1.293822	-2.653346	-2.159888
29	6	0	0.087579	-3.786257	0.000190
30	1	0	0.668305	-4.713372	0.000732
31	1	0	-0.548370	-3.797381	0.890437
32	1	0	-0.547589	-3.797987	-0.890605
33	6	0	1.919304	-2.760830	1.269117
34	1	0	2.734525	-2.039326	1.331422
35	1	0	1.292964	-2.652830	2.160477
36	1	0	2.368818	-3.759717	1.285142
37	6	0	-3.361783	-0.099873	-0.000186
38	6	0	-3.869304	0.625171	1.253291
39	1	0	-3.540639	1.668019	1.277780
40	1	0	-4.964609	0.617596	1.276767
41	1	0	-3.502225	0.132083	2.158858
42	6	0	-3.935179	-1.516951	-0.000386
43	1	0	-3.628567	-2.077272	-0.889146
44	1	0	-3.628777	-2.077554	0.888269
45	1	0	-5.028636	-1.466734	-0.000463
46	6	0	-3.869476	0.625416	-1.253428
47	1	0	-4.964796	0.617732	-1.276782
48	1	0	-3.540997	1.668323	-1.277791
49	1	0	-3.502462	0.132550	-2.159142

TS

mp2=full/6-31g(d)
EUMP2 = -1079.6230555585 AU
Thermal correction to Gibbs Free Energy = 0.390202

Center Number	Atomic Number	Atomic Type	Coordinates (Angstroms) X	Y	Z
1	15	0	-2.820942	0.464973	0.244029
2	6	0	-3.864108	-0.718443	-0.283656
3	6	0	-1.012451	0.172162	0.034484
4	6	0	-0.160947	1.317380	0.001472
5	6	0	-0.453729	-1.137703	0.005660
6	6	0	1.224388	1.101127	-0.010925
7	6	0	0.938952	-1.258458	0.003414
8	6	0	1.801898	-0.164472	0.003969
9	1	0	1.881258	1.956009	-0.031848
10	1	0	1.379040	-2.245822	-0.008753
11	6	0	-0.650370	2.782442	-0.033049
12	6	0	-1.283304	-2.433143	-0.007199
13	6	0	-1.904361	-2.697887	1.372886
14	1	0	-2.538150	-1.877248	1.706863
15	1	0	-2.514569	-3.607794	1.346083
16	1	0	-1.105464	-2.841341	2.106853
17	6	0	-2.332128	-2.395218	-1.111845
18	1	0	-2.729065	-3.395059	-1.324994
19	1	0	-3.345624	-1.880939	-0.841542
20	1	0	-1.972255	-1.926428	-2.031263
21	6	0	-0.421738	-3.665961	-0.339209
22	1	0	0.087262	-3.564632	-1.302676
23	1	0	0.322902	-3.866643	0.435588
24	1	0	-1.072275	-4.544165	-0.389576
25	6	0	-1.581837	3.027644	-1.232084
26	1	0	-2.523552	2.482631	-1.168960
27	1	0	-1.825800	4.094065	-1.293554
28	1	0	-1.081089	2.738034	-2.161840
29	6	0	0.511332	3.772647	-0.216464
30	1	0	0.095728	4.783062	-0.274208
31	1	0	1.209244	3.757972	0.625731
32	1	0	1.067022	3.590162	-1.141237
33	6	0	-1.322832	3.175657	1.293272
34	1	0	-2.232942	2.613624	1.501911
35	1	0	-0.626620	3.020416	2.123898
36	1	0	-1.590060	4.238122	1.266469
37	6	0	3.307596	-0.394257	0.007805
38	6	0	3.690361	-1.198497	1.257024
39	1	0	3.200637	-2.176318	1.273052
40	1	0	4.773096	-1.364488	1.284125
41	1	0	3.401577	-0.659740	2.164917
42	6	0	4.096510	0.915015	0.020735
43	1	0	3.890503	1.520227	-0.867546
44	1	0	3.871194	1.513112	0.909181
45	1	0	5.168718	0.694224	0.030875
46	6	0	3.701315	-1.181554	-1.248817
47	1	0	4.784231	-1.347519	-1.268857
48	1	0	3.211220	-2.158805	-1.282202
49	1	0	3.420689	-0.630202	-2.151754

2

mp2=full/6-31g(d)

EUMP2 = -1079.7940786280 AU

Thermal correction to Gibbs Free Energy = 0.396969

Center Number	Atomic Number	Atomic Type	Coordinates (Angstroms) X	Y	Z
1	15	0	-2.821328	0.519826	0.174681
2	6	0	-3.445835	-0.926317	-0.401899
3	6	0	-1.010579	0.229915	0.022431
4	6	0	-0.102264	1.330000	0.000995
5	6	0	-0.494815	-1.097232	-0.013227
6	6	0	1.273048	1.053840	-0.011515
7	6	0	0.886527	-1.294094	-0.007865
8	6	0	1.796298	-0.237326	-0.007612
9	1	0	1.967606	1.879135	-0.026417
10	1	0	1.271854	-2.306782	-0.015599
11	6	0	-0.535051	2.810327	-0.014064
12	6	0	-1.397809	-2.331346	0.022667
13	6	0	-1.790338	-2.624616	1.476772
14	1	0	-2.309195	-1.780595	1.935972
15	1	0	-2.446129	-3.502139	1.526636
16	1	0	-0.891025	-2.832721	2.064847
17	6	0	-2.640016	-2.098608	-0.846514
18	1	0	-3.256555	-3.006740	-0.831892
19	1	0	-4.533750	-1.000887	-0.426540
20	1	0	-2.311983	-1.959036	-1.889121
21	6	0	-0.710047	-3.579450	-0.541992
22	1	0	-0.305423	-3.395041	-1.542180
23	1	0	0.098524	-3.937638	0.100666
24	1	0	-1.442709	-4.390219	-0.613599
25	6	0	-1.438678	3.105097	-1.222119
26	1	0	-2.394324	2.582365	-1.177707
27	1	0	-1.653246	4.178808	-1.269120
28	1	0	-0.933131	2.816611	-2.149635
29	6	0	0.661471	3.763937	-0.152843
30	1	0	0.283821	4.790426	-0.188703
31	1	0	1.345397	3.698844	0.698504
32	1	0	1.225349	3.586356	-1.073647
33	6	0	-1.226672	3.193484	1.304639
34	1	0	-2.160709	2.657234	1.470172
35	1	0	-0.559247	2.989607	2.148383
36	1	0	-1.454880	4.265614	1.302181
37	6	0	3.291122	-0.530288	-0.009202
38	6	0	3.644337	-1.349783	1.238733
39	1	0	3.111525	-2.304664	1.257417
40	1	0	4.718966	-1.563265	1.261653
41	1	0	3.383053	-0.798082	2.147229
42	6	0	4.136290	0.743371	-0.000370
43	1	0	3.950441	1.357642	-0.886845
44	1	0	3.942215	1.350056	0.889583
45	1	0	5.198123	0.476414	0.003029
46	6	0	3.645817	-1.334410	-1.266699
47	1	0	4.720407	-1.548051	-1.290966
48	1	0	3.112084	-2.288471	-1.297842
49	1	0	3.386020	-0.771190	-2.168568

Me–P=C:

b3lyp/6-31g(d)
E(RB3LYP) = -419.194053996 A.U.

Standard orientation:

Center Number	Atomic Number	Atomic Type	Coordinates (Angstroms) X	Y	Z
1	6	0	0.000000	0.000000	-1.598461
2	1	0	0.000000	1.036680	-1.942378
3	1	0	0.897791	-0.518340	-1.942378
4	1	0	-0.897791	-0.518340	-1.942378
5	15	0	0.000000	0.000000	0.275275
6	6	0	0.000000	0.000000	1.881464

Rotational constants (GHZ): 155.5325070 5.7502891 5.7502891

TD-SCF

2

```
td=(nstates=10) cam-b3lyp/dgdzvp
Excited State   1:      Singlet-A      4.2842 eV  289.40 nm  f=0.1624  <S**2>=0.000
      77 -> 80        -0.25953
      79 -> 80         0.64455
```

Mes*P=CH_2

```
td=(nstates=10) cam-b3lyp/dgdzvp
Excited State   1:      Singlet-A      4.2444 eV  292.11 nm  f=0.0139  <S**2>=0.000
      77 -> 81        -0.28532
      80 -> 81         0.63656
```

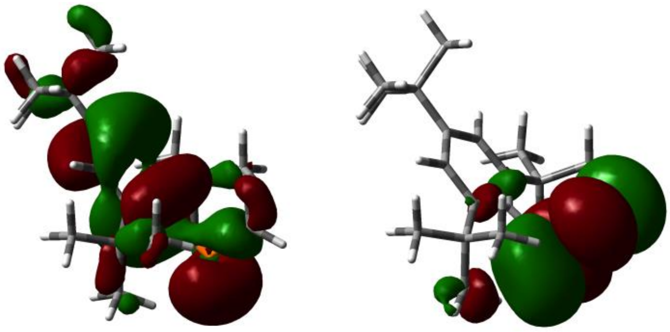

HOMO (left) and LUMO (right) of Mes*P=CH_2
